# Individual differences in processing resources modulate bimanual interference in pointing

**DOI:** 10.1007/s00426-018-1050-3

**Published:** 2018-07-17

**Authors:** Constanze Hesse, Laura Koroknai, Jutta Billino

**Affiliations:** 1 School of Psychology, University of Aberdeen, King’s Campus, Aberdeen, AB24 3FX UK; 2 Department of Experimental Psychology, Justus-Liebig Universitaet Giessen, Giessen, Germany

## Abstract

Coordinating both hands during bimanual reaching is a complex task that can generate interference during action preparation as often indicated by prolonged reaction times for movements that require moving the two hands at different amplitudes. Individual processing constraints are thought to contribute to this interference effect. Most importantly, however, the amount of interference seems to depend considerably on overall task demands suggesting that interference increases as the available processing resources decrease. Here, we further investigated this idea by comparing performance in a simple direct cueing and a more difficult symbolic cueing task between three groups of participants that supposedly vary in their processing resources, i.e., musicians, young adults and older adults. We found that the size of interference effects during symbolic cueing varied in the tested groups: musicians showed the smallest and older adults the largest interference effects. More importantly, a regression model, using processing speed and processing capacity as predictor variables, revealed a clear link between the available processing resources and the size of the interference effect during symbolic cueing. In the easier direct cueing task, no reliable interference was observed on a group level. We propose that the susceptibility to bimanual interference is modulated by the task-specific processing requirements in relation with the available processing resources of an individual.

## Introduction

Coordinating our two hands is an essential human skill that allows us to perform most everyday tasks, such as dressing ourselves, making breakfast, driving to work, or typing out a manuscript, with ease. Nevertheless, we have all experienced that there are certain limits to the independence with which we can move our two hands—for example, when trying to simultaneously rub our belly and tap our chest or when attempting to draw two different shapes with each hand (e.g., Albert & Ivry, 2009; Franz, Zelaznik, & McCabe, 1991).

Numerous studies have investigated these bimanual coordination constraints in the lab and have confirmed that there is a general preference of the motor system to synchronise bimanual movements temporally and align them spatially. In particular, Kelso and colleagues (e.g., Kelso, Putnam, & Goodman, 1983; Kelso, Southard, & Goodman, 1979a; Kelso, Southard, & Goodman, 1979b) provided seminal insights into bimanual coordination by showing that movement times of both hands tend to converge even if the requirements for the separate movements deviate substantially. According to Fitts’ law (Fitts, 1954), movements with longer amplitudes or with higher accuracy demands will take more time to execute. However, during bimanual reaching, the hand that performs a shorter or easier movement tends to extend its movement time to closely match the movement duration of the hand that performs a longer or harder movement. In addition to the coordination constraints that affect movement execution, it has also been found that movement planning times are often prolonged when movements are spatially incongruent (e.g., Blinch et al., 2014; Heuer, 1986; Heuer & Klein, 2006; Heuer, Spijkers, Kleinsorge, van der Loo, & Steglich, 1998; Spijkers, Heuer, Kleinsorge, & van der Loo, 1997). In other words, it seems to take participants longer to plan and initiate movements that require them to move their two hands to targets presented at different distances and/or directions as compared to starting movements to targets that are located equidistantly and in the same movement direction. This interference effect seems to indicate that incongruent movements are more complex to plan and, therefore, require longer processing times (Spijkers et al., 1997).

Two different explanations have been discussed in the literature for why reaction time (RT) costs occur for spatially incongruent movements. Originally, it was suggested that the effect is primarily due to planning constraints on the motor level (Heuer, 1993; Heuer et al., 1998; Spijkers & Heuer, 1995; Spijkers et al., 1997; Stelmach, Amrhein, & Goggin, 1988). That is, when two different parameters (such as movement amplitude) have to be concurrently specified during action planning neural cross-talk occurs and causes mutual inhibition in the processes of movement specification and thereby the observed interference effect (i.e., transient coupling hypothesis, see also Heuer, 1993; Spijkers & Heuer, 1995; Spijkers, Heuer, Steglich, & Kleinsorge, 2000). However, this view was called into question by a study by Diedrichsen and colleagues (2001). In this study, it was varied how the final movement targets were specified. Target locations for each hand were either specified by symbolic cues (such as letters) or by direct spatial cues that identified target locations by a sudden onset of the two movement targets. Prolonged movement initiation times for incongruent movements were only observed in conditions in which the movements were cued symbolically, but not when a direct cueing paradigm was used. Since the previous studies that found spatial interference effects when planning bimanual reaches had also employed symbolic cueing (e.g., Spijkers et al., 1997; Spijkers et al., 2000), Diedrichsen and colleagues (2001) suggested that the RT increase for incongruent movements is a result of the higher processing demands on response selection rather than of the higher complexity of planning and programming multiple motor commands. Thus, they concluded that interference occurs primarily on a cognitive level (i.e., at the stages of cue translation and response selection) rather than on a motor level (see also Hazeltine, Diedrichsen, Kennerley, & Ivry, 2003 for similar argument).

While there is still some debate about both explanations, most recent studies indicate that in fact, both processes contribute to the interference effect (Blinch et al., 2014; Blinch, Cameron, Franks, Carpenter, & Chua, 2015; Diedrichsen, Grafton, Albert, Hazeltine, & Ivry, 2006; Heuer & Klein, 2006). However, interference on the motor level seems comparatively smaller as compared to the cognitive interference arising during symbolic cueing. The reason for proposing the existence of two independent, but additive processes is primarily related to the observation that small but robust spatial interference effects on movement initiation times can be found even when direct cueing paradigms are employed (e.g., Blinch et al., 2014, 2015; Diedrichsen et al., 2006; Heuer & Klein, 2006).

In a recent study, Stanciu, Biehl, and Hesse (2017) hypothesised that there might, after all, be a common cause that determines whether or not an interference effect occurs during bimanual movement preparation. Specifically, they argued that the symbolic cueing condition constitutes a dual-task situation that requires participants to identify the cues and to subsequently link them to the correct motor response, which in turn requires them to keep the specific mapping rules in working memory when doing the task. Direct cueing eliminates this cue–response translation process and, therefore, requires overall less cognitive resources than a symbolic cueing task. Hence, Stanciu et al. (2017) proposed that the occurrence and the size of the interference effect may depend on the overall task demands and, therefore, the amount of central resources available for movement planning. Support for this notion came from the observation that interference effects were nullified in a symbolic cueing condition when cue–response compatibility was maximised (using arrows as cues). This is in line with other studies indicating that the interference effect decreases as the translational load is reduced (Kunde & Weigelt, 2005; Weigelt, Rieger, Mechsner, & Prinz, 2007; Wenderoth & Weigelt, 2009). Furthermore, Stanciu and colleagues showed that interference effects for movements with different spatial requirements can reliably be observed in direct cueing conditions when a resource-demanding secondary task (i.e., attention or memory task) is introduced. The fact that interference occurred even when the secondary task was completely unrelated to the primary movement task provided first empirical support for the assumption that limiting the central processing resources available during movement planning may indeed be sufficient to evoke a bimanual interference effect.

Here, we wanted to further test the suggestion that the bimanual interference effect for planning movements with different spatial requirements is linked to the overall task demands and the available processing resources. If this proposition is true, then individuals that vary in their available resources should also vary in the amount of bimanual interference they show. To test this hypothesis, we aimed to investigate bimanual interference in congruent and incongruent bimanual reaching tasks in three specific groups, i.e., younger adults with musical expertise, younger adults, and older adults. The assumption underlying our study is that the processing resources available to perform the tasks differ for the three participant groups, meaning that they should show differences in their vulnerability to interference effects. This assumption is of course not plucked out of thin air, but founded on a rich empirical base.

Musical expertise has been repeatedly associated with superior functional capacities in motor and cognitive domains. It has been shown that musicians who have extensive experience from a young age in playing an instrument that imposes similar workload on both hands (such as the piano or woodwind instruments) not only show superior bimanual coordination performance (e.g., Hughes & Franz, 2007; Jäncke, Schlaug, & Steinmetz, 1997; Verheul & Geuze, 2004), but also require less cortical effort to plan and execute such movements (Haslinger et al., 2004). That is, as a result of their long-term practice and training motor preparation becomes more efficient as indicated by a recruitment of smaller cortical networks (e.g., Haslinger et al., 2004; Jäncke, Shah, & Peters, 2000). Interestingly, expertise in independent finger movements in musicians does not seem to be linked to enhanced interhemispheric inhibitory control (Nordstrom & Butler, 2002; Ridding, Brouwer, & Nordstrom, 2000). Training studies have suggested that intensive motor practice might induce plastic changes that favour a constructive interaction of hemispheres rather than a mutual inhibition during task execution (Hortobágyi et al., 2011; Shim et al., 2005). Overall, it has been argued that musicians, or more generally individuals with an increased expertise in bimanual coordination tasks, require less effort to plan efficient bimanual hand movements. Thus, critical resources are freed and are putatively available to focus on other aspects, such as artistic expression, during performance (see Krings et al., 2000 for a similar argument). Beyond advantages in motor processing enhanced performance on a variety of cognitive measures has been described in musicians. In particular, musicians have been found to outperform non-musicians with regard to working memory (George & Coch, 2011; Hansen, Wallentin, & Vuust, 2013), spatial cognition (Sluming, Brooks, Howard, Downes, & Roberts, 2007), and executive control capacities (Zuk, Benjamin, Kenyon, & Gaab, 2014). In summary, we propose that young adults with musical expertise represent a group that can be plausibly assumed to have an advantage in processing resources that contribute to the execution of complex motor coordination tasks.

In contrast, age-related decline in functional systems is likely to result in substantial disadvantages during bimanual coordination tasks. Numerous studies have described reduced motor control capacities in older adults (e.g., Leversen, Haga, & Sigmundsson, 2012; Seidler et al., 2010; Ward & Frackowiak, 2003). Specifically, it has been shown that bimanual movements slow down and become less accurate with increasing age (for recent reviews see: Krehbiel, Kang, & Cauraugh, 2017; Maes, Gooijers, de Xivry, Swinnen, & Boisgontier, 2017). Furthermore, older adults have increased difficulties performing asymmetric movements (Stelmach et al., 1988) and inhibiting preferred coordination patterns (Swinnen, 1998). Finally, it has been reported that motor performance deteriorates more strongly when older adults have to share attentional resources between a movement task and secondary cognitive tasks (Huxhold, Li, Schmiedek, & Lindenberger, 2006; Lee, Wishart, & Murdoch, 2002). Based on an extensive review of the current literature, Maes et al. (2017) came to the conclusion that aging relates to an increased involvement of cognitive processes during bimanual coordination, meaning that older adults require more attention to do the same task equally well as younger adults. Hence, if task complexity and difficulty are increased, control deficits become more noticeable in older adults as their executive resources are exceeded more quickly (Lee et al., 2002; Wishart, Lee, Murdoch, & Hodges, 2000). Considering in addition the well-established knowledge on overall declining cognitive control resources in old age (e.g., Hasher & Zacks, 1988; Park & Reuter-Lorenz, 2009; Salthouse, 1996; West, 1996), it can be expected that bimanual coordination is critically challenged by limited processing resources in older adults.

In a nutshell, the aim of the current study is to examine the occurrence of the bimanual interference effect during movement planning in three different participant groups for which substantial differences in processing resources can be assumed. Since in motor coordination tasks, the functional contributions of motor and cognitive resources are closely interlinked, we do not intend to differentiate between specific resources in detail. Specifically, we hypothesise that bimanual interference effects scale with the overall available processing resources. Individuals with more resources (e.g., due to musical expertise) are expected to be relatively less prone to interference effects than individuals with less resources (e.g., due to age-related changes). In addition, individual differences should be more pronounced in more difficult tasks which pose higher demands (i.e., symbolic cueing as compared to direct cueing) and are thus likely to be affected more strongly by reduced resources.

## Methods

### Participants

We investigated three participant groups that are assumed to differ in their available resources. We recruited a group of 16 young adults with no instrumental expertise (non-musicians), a group of 16 young musicians, and a group of 16 older adults with no instrumental expertise and training. The young adult group consisted of undergraduate and postgraduate students of the University of Aberdeen (age range 19–25 years, mean age 22 years, 5 male) who did not actively play an instrument and have had no formal instrumental training or education. Two of the young adults were left-handed by self-report. The musicians group was also recruited from within the University of Aberdeen student population (age range 18–27 years, mean age 21 years, 4 male, 4 left-handed by self-report). All musicians had played their instrument for at least 7 years (mean 11 years, std 3.5 years). Fourteen of the musicians played the piano, one saxophone and clarinet, and one played the drums. All but two of the participants still engaged in regular weekly practice. The 16 older adults were recruited using the University of Aberdeen, School of Psychology’s participant panel (age range 64–77 years, mean age 69.5 years, 7 males, 2 left-handed by self-report). Older adults did not report to have instrumental expertise or training. Any history of ophthalmologic and neurologic disorders as well as medications presumed to interfere with motor functioning were screened out by a detailed interview protocol. All participants had normal or corrected-to-normal vision. Furthermore, older adults had also been screened for mild cognitive impairment (cut-off score of ≥ 26 on the Montreal Cognitive Assessment scale) maximally 6 months prior to participating in our study.

All participants provided written informed consent prior to participating in the study and the protocol was approved by the ethics committee of the School of Psychology at the University of Aberdeen.

### Setup and stimuli

**Fig. 1:**
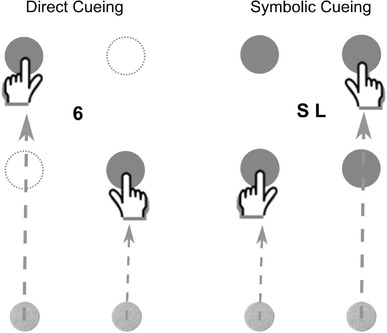
Illustration of cueing conditions: left panel: LS movement in the direct cueing condition, right panel: short–long (SL) movement in the symbolic cueing condition

The setup was identical to the one we have used and described previously (Stanciu et al., 2017). Participants sat in a dimly lit room in front of a monitor (19″ IPS monitor, Dell P1914S, 1280 × 1024 pixel, 30 cm × 37.5 cm, refresh rate 60 Hz) that was flatly screwed onto the table surface in vertical orientation (portrait mode). The screen was protected by a fitted Plexiglas panel (3 mm in thickness). The start positions for the left and right index fingers were marked by two felt pads (diameter of 10 mm) that were attached to the lower edge of the monitor frame at a distance of 4.5 cm from the central line (i.e., 9 cm distance between the two felt pads). The pointing targets were displayed on the monitor (forming an imaginary central rectangle) and placed straight ahead and in front of the fingers’ start positions. The near target was displayed at a distance of 13.5 cm from the start position and the far target at a distance of 25.5 cm (see Fig. 1). All target positions were filled black circles with a diameter of 12 mm presented on a grey background.

Hand movements were captured at a sampling rate of 200 Hz using an infrared optoelectronic motion tracker (Optotrak 3020, Northern Digital Incorporation, Waterloo, Ontario, Canada). Small infrared markers were attached to the nails of the index finger of each hand using Blu Tack. The experiment was programmed in MATLAB using the Psychophysics Toolbox (Brainard, 1997; Kleiner, 2010) and the custom-built Optotrak Toolbox (Franz, 2004).

### Procedure

Our procedure comprised two established bimanual reaching tasks that differ in complexity (see Fig. 1). Specifically, we used (a) a direct cueing paradigm in which reaching targets are indicated directly by an offset of the movement irrelevant target locations and (b) a symbolic cueing paradigm that requires cue–response translation based on symbolic cues to identify the relevant movement targets. Both reaching conditions were blocked and counterbalanced across participants in each group. Within each block, we varied the congruency of the bimanual movements (congruent movements with either short or long amplitudes vs. incongruent movements with different amplitudes for each hand). The procedure yielded four different movement patterns. There were two possible symmetric movement patterns, where participants had to move both hands to equidistant targets on the left- and right-hand sides, respectively (i.e., both hands reaching for either a near or a far targets) and two possible asymmetric movement patterns, where participants had to cover different movement amplitudes with both hands (i.e., the left hand reaching for a near target and the right hand reaching for a far target or vice versa). Note that the left hand was always required to point to a left-hand side target and the right hand to a right-hand side target, meaning that the hands never crossed. The different movement patterns were selected randomly and repeated ten times within each block resulting in a total of 40 trials per cueing condition. Each block was preceded by four practice trials to familiarise participants with the task.

At the beginning of each trial, the experimenter ensured that participants placed their index fingers on the start positions. The trial was then started manually by the experimenter with a key press and all four movement targets were displayed for a preview period of 2500 ms on the screen. In the direct cueing condition, two of the movement targets were removed from the screen after the preview period (one on either side of the midline), leaving only the targets visible participants were supposed to point to. In the symbolic cueing condition, all four movement targets remained visible after the preview period, but centrally, two letters were displayed which cued the pointing targets. The left letter specified the movement amplitude for the left hand and the right letter the amplitude for the right hand (i.e., “SS” and “LL” for symmetric short and long movement amplitudes, respectively, and “SL” and “LS” for the two asymmetric movement conditions). Each letter was 10 mm in size (line width 1.5 mm) and there was a 5 mm space between them. This symbolic cueing condition introduces an additional cognitive demand, namely, the translation of symbols into the spatial positions of the pointing targets. Note that in the direct cueing condition, we opted for identifying targets by a sudden offset of the two movement irrelevant locations rather than a sudden onset of the two relevant targets. This was done to ensure maximum similarity between the direct cueing and the symbolic cueing condition in which also all four possible movement targets were displayed before the movement was cued hence allowing partial pre-programming (see Hazeltine et al., 2003). Furthermore, it has been argued that symbolic cueing requires participants to pay attention to an additional location (i.e., the centre of the display) which in turn may subtract attention from the movement targets and subsequently increase the interference effect in this condition (Hazeltine et al., 2003). Again, to keep condition as comparable as possible, we introduced a display of random digits (between 1 and 9) in the centre of the display during direct cueing. Digits were presented at a size of 10 mm and were displayed for 150 ms (9 frames) with a blank interval of 200 ms (12 frames) between presentations. Participants were asked to fixate on the digits, but told that they were irrelevant for the task.

In both conditions, participants were instructed to point to the cued targets as quickly and accurately as possible, using their index fingers. In all trials, participants had 2 s to complete their movements to the targets after the movements were cued. One older adult (male) was excluded from further data analysis as his reaction times frequently exceeded the response interval provided.

### Data analysis

To determine reaction time (RT), we computed the resultant velocity of the positional data obtained from both infrared markers. Movement onset and offset were defined by a velocity threshold (all calculations identical to Stanciu et al., 2017). The moment the markers exceeded a velocity of 0.05 m/s was defined as movement onset and the moment their velocity dropped below 0.05 m/s, and their distance was less than 20 mm from the movement target, was defined as movement offset. The time between the cueing of the movement targets and movement onset was defined as RT and the time between movement onset and movement offset as movement time (MT). Trials were excluded if RT and movement offset could not reliably been identified using those criteria and when there was more than 100 ms difference between the RTs of the left and right hands (older adults: 1.25%; young adults: 0.20%; young musicians: 0% of all trials). For statistical analysis, median RTs were calculated for each condition and participant. Consistent with our previous work (Stanciu et al., 2017), we considered medians as a more robust measure of reaction times than means (note that both mean and median values are provided at the linked online data repository). Median RTs were collapsed across both hands and averaged across symmetric movement conditions (short–short and long–long) and asymmetric movement conditions (short–long and long–short). As a measure of movement accuracy, we determined the average distance error indicating the absolute (unsigned) Euclidean distance between the target and the finger positions at movement offset. The accuracy and MT data were reduced equivalently to the RT data. If not stated otherwise, data was analysed using mixed ANOVAs with the within-subject factors cueing condition (direct cueing vs. symbolic cueing) and movement congruency (symmetric vs. asymmetric movements) and the between subject factor group (young musicians vs. young adults vs. older adults). Post-hoc tests were Bonferroni–Holmes corrected for multiple comparisons if applicable. All values are presented as means ± SEMs. A significance level of *α* = 0.05 was used for all statistical analysis. The data set is available at zenodo.org, 10.5281/zenodo.1207940.

## Results

### Reaction times

**Fig. 2:**
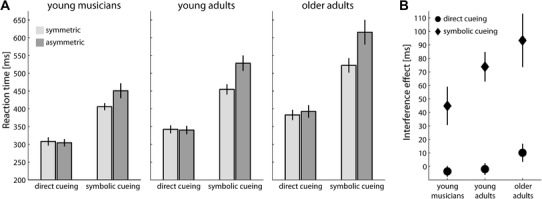
Reaction times. **a** Average RTs in each participant group as a function of cueing condition and movement congruency. **b** Interference effects in each participant group and cueing condition, respectively. The interference effect is given by the average RT difference between incongruent and congruent movements. Error bars depict ± 1 SEM between subjects

We were initially interested in determining whether movement planning times vary systematically between the three participant groups and between cueing conditions that involve differential task demands. To this end, we conducted a 2 × 2 × 3 mixed ANOVA on RTs with the within-subject factors cueing condition (direct cueing vs. symbolic cueing) and movement congruency (symmetric vs. asymmetric movements) and the between subject factor *group* (young musicians vs. young adults vs. older adults). Figure 2a shows the average RTs in each group as a function of cueing condition and movement congruency.

We found significant main effects for the within-subject factors cueing condition, *F*(1, 44) = 263.10, *p* < .001, $\eta _{{\text{p}}}^{2}$ =.86, and movement congruency, *F*(1, 44) = 57.46, *p* < .001, $\eta _{{\text{p}}}^{2}$ = .57. However, these main effects were qualified by several interactions. First, there was a significant interaction between both within-subject factors, *F*(1, 44) = 59.53, *p* < .001, $\eta _{{\text{p}}}^{2}$ = .58. Separate post-hoc ANOVAs for each cueing condition showed that there was no effect of movement congruency on RT in the direct cueing condition (symmetric movements: 344 ± 7 ms vs. asymmetric movements: 346 ± 8 ms, *p* = .62), while the movement congruency effect in the symbolic cueing condition was highly significant (symmetric: 461 ± 9 ms vs. asymmetric: 532 ± 15 ms, *p* < .001). In other words, the size of the interference effect, calculated as the RT difference between asymmetric and symmetric movements, was about 2 ms in the direct cueing condition and 71 ms in the symbolic cueing condition (see also Fig. 2b).

In addition, the initial ANOVA also confirmed a significant main effect of the between subject factor group, *F*(2, 44) = 13.90, *p* < .001, $\eta _{{\text{p}}}^{2}$ = .39, indicating overall differences in RTs between participant groups (musicians: 367 ± 15 ms, young adults: 416 ± 15 ms, older adults: 478 ± 15 ms, all p < .022). Furthermore, there were also significant interaction effects between group and cueing condition, *F*(2, 44) = 3.31, *p* = .046, $\eta _{{\text{p}}}^{2}$ = .13, as well as group and movement congruency, *F*(2, 44) = 3.53, *p* = .038, $\eta _{{\text{p}}}^{2}$ = .14. The three-way interaction was not significant (*p* = .26).

Post-hoc analyses investigating the interaction effect between group and cueing condition revealed that RT differences between participant groups were slightly less pronounced in the direct cueing condition than in the symbolic cueing condition ($\eta _{{\text{p}}}^{2}$ = .31 vs. $\eta _{{\text{p}}}^{2}$ = .35). In both cueing conditions, direct and symbolic, young musicians consistently showed that the fastest RTs and older adults showed the longest RTs. In the direct cueing condition, older adults were significantly slower in initiating their movements than both younger adult groups (81 ± 18 ms slower than musicians, *p* < .001, and 46 ± 18 ms slower than non-musicians, *p* = .014). The difference between the two young groups approached significance (mean difference 35 ± 18 ms, *p* = .057). In the symbolic cueing condition, older adults reacted even more slowly than the two younger participant groups (141 ± 29 ms slower than musicians, *p* < .001, and 77 ± 29 ms slower than non-musicians, *p* = .01). In this condition, the RT difference between musicians and young adults increased to 63 ± 28 ms and was statistically significant, *p* = .03.

The size of the interference effects for the different groups and conditions is shown in Fig. 2b which also illustrates the significant interaction effect between group and movement congruency. Across both cueing conditions, young musicians showed the smallest interference effect (21 ± 8 ms), followed by young adults (36 ± 8 ms) and then older adults (52 ± 8 ms). However, only the difference between young musicians and older adults reached statistical significance (*p* = .011, both other *p*s > .19)

**Fig. 3:**
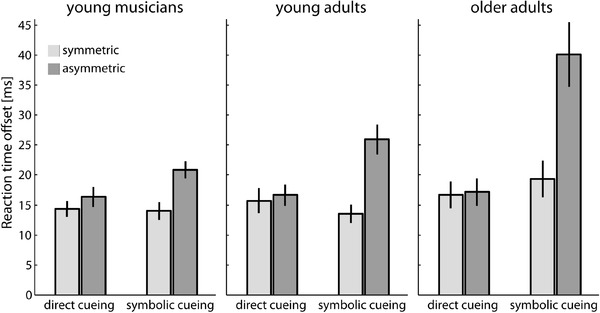
Average RT offsets between both hands in each participant group as a function of cueing condition and movement congruency. Error bars depict ± 1 SEM between subjects

Since the pre-analysis of our data suggested possible group differences in the synchronicity of the movement onsets for both hands (see “Methods”), we aimed to clarify whether RT offsets also varied systematically with cueing condition and movement congruency. Condition-dependent differences may be indicative of strategic differences applied by individuals to deal with the task and may hence mediate the observed interference effects. For each participant, we computed the median offset between the RTs for the two hands. Figure 3 illustrates the mean RT offsets for each group, cueing condition and movement congruency condition.

A 2 (cueing condition) × 2 (movement congruency) × 3 (*group*) mixed ANOVA yielded significant results for all main and interaction effects (all *p*s ≤ .026). We followed up the three-way interaction, *F*(2, 44) = 7.86, *p* = .001, $\eta _{{\text{p}}}^{2}$ = .26, using two separate 2 (*movement congruency*) × 3 (*group*) mixed ANOVAs for each of the cueing conditions. In the direct cueing condition, RT offsets were affected neither by movement congruency nor by group (all *p*s ≥ .11). The average RT offset between the hands was about 15 ms. In contrast, we found significant main effects of movement congruency, *F*(1, 44) = 75.12, *p* < .001, $\eta _{{\text{p}}}^{2}$ = .63, and group, *F*(2, 44) = 6.56, *p* = .003, $\eta _{{\text{p}}}^{2}$ = .23, as well as a significant interaction effect between both factors, *F*(2, 44) = 6.76, *p* = .003, $\eta _{{\text{p}}}^{2}$ = .24, in the symbolic cueing condition. Overall, the RT offsets were larger for incongruent than for congruent movements and larger for older adults than for both younger groups. The interaction effect indicated that the RT-offset difference between congruent and incongruent movements was most pronounced for older adults (21 ms) and least pronounced for musicians (7 ms) with young adults falling in-between (12 ms). These findings further support the notion that older adults show an increased vulnerability to bimanual interference, in particular in the symbolic cueing condition, and that this is not only indicated by overall prolonged RTs, but also by an increased asynchrony in the RTs of both hands.

Based on these findings, we wanted to further explore the association between individual differences in the size of the interference effect and the available processing resources. Two potential predictors providing appropriate processing measures were identified: (1) RTs for symmetric movements in the direct cueing condition provide a baseline measure and can thus be considered as an indicator of general processing speed and (2) RT increase (costs) in the symbolic cueing condition as compared to the direct cueing condition when performing symmetric movements provides a measure for an individual’s vulnerability to additional task demands and can thus be considered an indicator of the available processing capacity. Please note that the labelling of both predictor variables as proxies for general processing speed and processing capacity is tentative as we did not determine both measures with independent tests. Keeping this in mind, the labels can, however, be considered appropriate descriptions of our parameters.

**Fig. 4:**
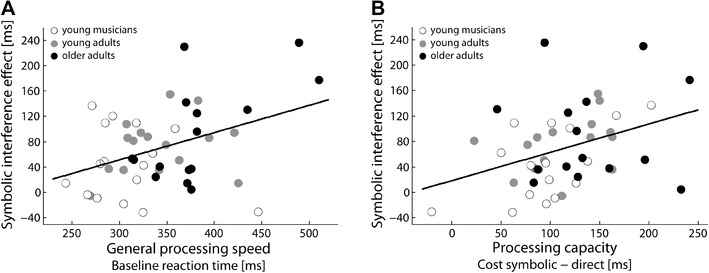
Association between processing capacities and the interference effect in the symbolic cueing condition. **a** Correlation between reaction times in the symmetric direct cueing condition, used as proxy for general processing speed, and the interference effect. **b** Correlation between RT costs caused by cue–response translation, used as proxy for processing capacity, and the interference effect

In the direct cueing condition, the magnitude of the interference effects was marginal and variance between individuals was limited. Consequently, correlations between bimanual interference and both resource measures did not reach significance (*r*(47) = 0.15, *p* = .332 for general processing speed; *r*(47) = 0.27, *p* = .06 for processing capacity). However, we found strong correlations in the symbolic cueing condition in which pronounced interference was observed. Figure 4 illustrates the link between the size of the interference effect and processing resources in the symbolic cueing condition. For general processing speed, we determined a correlation of *r*(47) = 0.40, *p* = .006, and for available processing capacity a correlation of *r*(47) = 0.37, *p* = .011. Figure 4 also depicts group membership for each data point. This illustration suggests that the reported correlations are not merely driven by group differences, but can actually be observed across the whole sample. Consistently, with this observation, a partial correlation analysis controlling for the factor group, yielded comparable results: *r*(44) = 0.38, *p* = .009 for general processing speed, and *r*(44) = 0.35, *p* = .006 for processing capacity.

Multiple linear regression analysis was used to test whether the interference effect can be predicted by individuals’ processing resources, i.e., processing speed and processing capacity as indicated by RT costs. Both predictor variables were uncorrelated, *r*(47) = −0.01, *p* = .926. Using the enter method, we found that the two predictors explained 30% of the variance in the magnitude of the interference effect, *F*(2, 44) = 9.35, *p* < .001, *R*^2^ = 0.30. Evaluation of the *β* coefficients showed that both predictors had a significant partial effect in the full model, *t*(44) = 3.19, *p* = .003 for processing speed and *t*(44) = 2.96, *p* = .005, for processing capacity. Overall, the regression analysis validates that individual differences in susceptibility to the interference effect critically depends on processing resources.

### Movement times

**Fig. 5:**
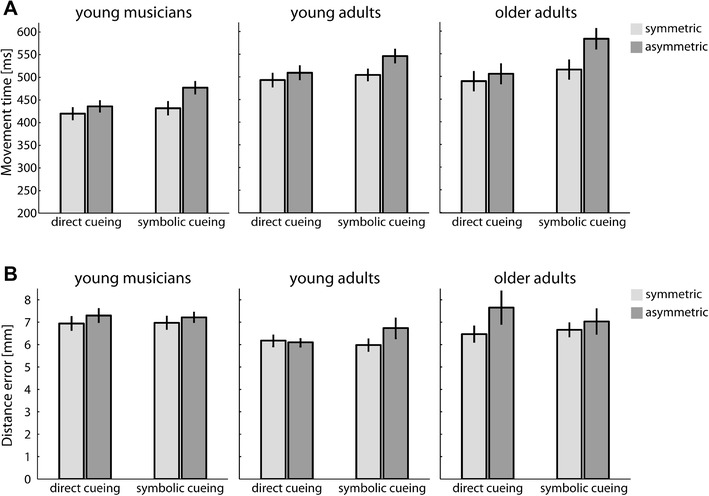
MTs and accuracy data. **a** Average MTs in each participant group as a function of cueing condition and movement congruency. **b** Average distance error in each participant group as a function of cueing condition and movement congruency. Error bars depict ± 1 SEM between subjects

It has been argued that one possible reason for why bimanual interference effects are not consistently observed might relate to differences in overall MTs. In particular, it has been speculated that if participants start moving their hand before movement amplitudes have been fully specified, and thus, motor programming occurs in flight, the motor interference due to amplitude specification may no longer be observable (Heuer & Klein, 2006). To test how our results on individual differences in the interference effect are affected by MTs in our three different participant groups, MTs were collapsed across both hands and averaged across symmetric movement conditions (short–short and long–long) and asymmetric movement conditions (short–long and long–short). Figure 5a provides an overview of the average MTs across all conditions and groups. Note that MTs calculated separately for long and short movements depending on the movement amplitude of the second hand are available at the online data repository associated with this article.

A 2 × 2 × 3 mixed ANOVA revealed main effects of cueing condition, *F*(1, 44) = 30.3, *p* < .001, $\eta _{{\text{p}}}^{2}$ = .41, movement congruency, *F*(1, 44) = 132.8, *p* < .001, $\eta _{{\text{p}}}^{2}$ = .75, as well as *group, F*(2, 44) = 7.03, *p* = .002, $\eta _{{\text{p}}}^{2}$ = .24. Post-hoc tests showed that MTs were significantly faster for musicians (441 ± 17 ms) than for both young adults (513 ± 17 ms) and older adults (523 ± 17 ms), both *p* ≤ .004. The difference in MTs between young and old adults was not significant (*p* = .67). The main effect of cueing condition indicated that MTs were overall quicker in the direct cueing condition (476 ± 10 ms) than in the symbolic cueing condition (509 ± 10 ms) and the main effect of congruency was caused by the fact that MTs tended to be about 34 ± 10 ms longer in the asymmetric conditions than in the symmetric ones. The main effects were further qualified by a significant two-way interaction between cueing condition and movement congruency, *F*(1,44) = 65.36, *p* < .001, $\eta _{{\text{p}}}^{2}$ = .60, indicating that the effect of movement congruency was more pronounced in the symbolic cueing condition as compared to the direct one (see Fig. 5a). There were no interaction effects between movement congruency and group as well as cueing condition and group (both *p* > .15). Finally, the three-way interaction between all factors also reached significance, *F*(2, 44) = 3.42, *p* = .042, $\eta _{{\text{p}}}^{2}$ = .14. This interaction indicates that MTs increased more strongly for older adults when asymmetric movements were required during symbolic cueing, while during direct cueing, the MT increase for asymmetric movements was similar for all three groups.

Overall, there is no indication that more pronounced interference effects in older adults were driven by relatively shorter movement times. Further supporting this notion, we found no correlation between overall MTs and the size of the bimanual interference effect in the symbolic cueing condition, *r*(47) = 0.17, *p* = .27, and even a positive correlation in the direct cueing condition, *r*(47) = 0.32, *p* = .027, indicating that participants with longer movement times actually tended to show larger congruency effects.

Finally, one might argue that potentially interference effects in RTs are confounded with an individual’s ability to decouple the movement execution times of both hands. In other words, the more individuals struggle to execute two movements with different spatial requirements the longer movement planning will take. To test for this possibility, we calculated for each participant the correlation between MTs of the left and right hand in all four movement conditions (SS, LL, LS, and SL). We then Fisher *z*-transformed the correlation coefficients and averaged them for the congruent (SS and LL) and incongruent (SL and LS) conditions. A 2 × 2 × 3 mixed ANOVA on the data revealed a main effect of cueing condition, *F*(1, 44) = 6.71, *p* = .013, $\eta _{{\text{p}}}^{2}$ = .13 and an interaction effect between cueing condition and movement congruency, *F*(2, 44) = 4.94, *p* = .031, $\eta _{{\text{p}}}^{2}$ = .10. All other main effects and interaction effects were not significant (all *ps* ≥ .06). Post-hoc analyses showed that in the direct cueing condition, MT coupling was, as expected, stronger for congruent movements (*z* = 0.89) than for incongruent movements (*z* = 0.41). However, this was not the case in the symbolic cueing condition in which MTs were coupled equally strongly in both congruency conditions (congruent: *z* = 0.91 vs. incongruent: *z* = 1.12). Most importantly, however, movement time coupling was not moderated by group. Thus, it seems unlikely that our observed group differences in interference effects in RTs can be attributed to differences in the ability to execute decoupled hand movements.

### Movement accuracy

To test if differences in movement planning times between the three tested groups affected the resulting movement accuracy, we determined the final movement error as the Euclidean distance between the fingers’ end positions and the final target position. Figure 5b illustrates similar distance errors across groups and cueing conditions. A 2 (cueing condition) × 2 (movement congruency) × 3 (group) mixed ANOVA on the data only revealed a significant main effect of movement congruency, *F*(1, 44) = 8.13, *p* = .007, $\eta _{{\text{p}}}^{2}$ = .16. Post-hoc tests showed that movement accuracy was slightly reduced for asymmetric movements (7.0 ± 0.2 mm) as compared to symmetric ones (6.5 ± 0.2 mm). None of the other main effects or interaction effects became significant (all *p* > .08). That is, all three participant groups showed similar movement accuracy as indicated by the distance errors. In addition, task demands as given by the different cueing conditions did not modulate movement accuracy.

## Discussion

In this study, we aimed to investigate how task demands in relation with the available processing resources modulate the bimanual interference effect, i.e., the finding that RTs tend to be prolonged for bimanual reaching movements with asymmetric amplitudes as compared to symmetric ones. The previous studies have shown that the size of the interference effect strongly depends on how these movements are cued. Specifically, interference effects were found to be larger and seem to be occurring more reliably when symbolic cueing conditions are employed. In contrast, during direct cueing, interference effects are strongly attenuated, or even disappear completely (Diedrichsen et al., 2006; Diedrichsen et al., 2001; Heuer & Klein, 2006).

Two independent mechanisms have been discussed in the literature to be responsible for the occurrence of the bimanual interference effect. First, interference was assumed to occur at the motor programming level as the simultaneous generation of two different motor commands may cause mutual inhibition (e.g., Heuer, 1993; Heuer et al., 1998; Spijkers et al., 1997). Second, interference can also occur at a cognitive level, in particular during symbolic cueing, where cue-translation processes are more complex for the selection and generation of asymmetric movements (Albert, Weigelt, Hazeltine, & Ivry, 2007; Diedrichsen et al., 2001; Diedrichsen, Ivry, Hazeltine, Kennerley, & Cohen, 2003; Hazeltine et al., 2003). Critically, for our study, while accounts differ on the question at which level the interference arises, they do share the assumption that increased RTs for incongruent movements can generally be linked to a higher task complexity (for review, see Wenderoth & Weigelt, 2009). In line with this notion, Stanciu et al. (2017) have recently shown that bimanual interference increases in direct cueing conditions when an additional (movement-independent) secondary task has to be performed and can be abolished in symbolic cueing conditions by minimising the translational load. Based on these findings, they concluded that it is not primarily the employed cueing procedure that determines the amount of bimanual interference, but instead, the overall task demands and thus the processing resources available for dealing with the task at hand.

In the current study, we further tested this hypothesis by investigating the role of available individual resources that are needed for complex motor coordination. Specifically, we compared three participant groups for which there is consistent evidence of pronounced differences in functional resources. In particular, young musicians can be assumed to show processing advantages as compared to young adults without musical training. Numerous studies have documented superior performance in motor and cognitive tasks in musicians (e.g., Hansen et al., 2013; Haslinger et al., 2004). In contrast, increasing age is associated with declining resources in a variety of functional systems (e.g., Leversen et al., 2012; Park & Reuter-Lorenz, 2009), so that processing disadvantages during motor coordination can be supposed. Our aim was not to differentiate between specific resources, i.e., specific cognitive or motor resources, but to consider overall interindividual differences in available processing resources as a critical modulating factor of the bimanual interference effect.

In accordance with the assumption that musicians show superior bimanual control (e.g., Hughes & Franz, 2007) and older adults show declined bimanual control (e.g., Stelmach et al., 1988), we observed large differences in the overall RTs and MTs between these two groups. That is, as expected, musicians were generally much faster to initiate and execute their movements than older adults. Most importantly, the groups also differed in the amount of bimanual interference they showed and the observed interference effect scaled with the available processing resources. In short, the novel and intriguing observations of our study are that (a) the size of the interference varied with the resourcefulness of our three participant groups in line with our predictions (i.e., smaller interference of musicians and largest interference for older adults) and (b) that on an individual data level, processing speed and processing capacity provided good predictors to estimate susceptibility to interference when planning movements with different spatial requirements.

Thus, our results provide evidence that differences in individual resources play a crucial role for the emergence of the interference effect. However, our data supported this link only for the symbolic cueing condition. In line with the previous literature, we found that interference effects were much more pronounced during symbolic cueing as compared to direct cueing. In fact, during direct cueing, none of the groups showed reliable interference effects, so that group differences eluded proper examination. Yet, it is worth noting that there was descriptively still an increase in the interference effect for older adults (10 ms, *p* = .08, see Fig. 2b). The hypothesis that the interference effect should be affected more strongly by interindividual resource differences in more demanding tasks was not supported by the group-based analysis (i.e., no three-way interaction effect in our RT analyses). We speculate that the relatively dominant effect of task demands might have obscured the hypothesised interaction effect. The correlational analysis of our continuous data, i.e., individual resources and interference effects, yielded an overall similar pattern in both cueing conditions. However, the size of the interference effect was significantly linked to processing resources in the more demanding, symbolic cueing condition only, but not in the direct cueing condition. This dissociation tentatively suggests that older adults are more vulnerable to bimanual interference in general and that interference effects might emerge eventually during direct cueing, as processing resources decline further. Some additional indirect support for this assumption comes from the observation that there was a positive correlation between the size of the interference effect during direct cueing and the overall MTs (*r* = .32, *p* = .027). Hence, participants who took longer to execute their movements, supposedly indicating a reduction in processing resources, were more likely to show an interference effect.

It has been argued previously that symbolic cueing conditions constitute a form of dual tasking as successful performance requires participants to correctly identify cues and retrieve the cue–response assignments from working memory before movement preparation can begin (Albert et al., 2007; Hazeltine et al., 2003). In addition, a recent study has identified the role of response selection processes (i.e., selecting and integrating the two different movements) as the primary source of the increased planning costs for bimanual asymmetric movements (Blinch, Franks, Carpenter, & Chua, 2018). As stimulus–response compatibility is reduced in the symbolic cueing condition, response selection becomes a much more demanding process than in the direct cueing condition. Thus, demanding processes under symbolic cueing conditions take up limited resources and hence reduce the processing capacity available to prepare the intended movement.

Furthermore, it has been proposed that older adults are able to perform as well in bimanual coordination tasks as younger adults, but that they need to allocate more attention to the task to maintain a comparable level of performance (i.e., attention–allocation hypothesis, see Lee et al., 2002). Accordingly, an age-related drop in performance can usually only be observed when the task demands exceed the resources available to an individual. In relation to our findings, this would mean that the direct cueing task constitutes quite an easy task, and even though the groups might supposedly vary in the amount of effort they had to put in, they all seemed to still possess sufficient resources to accomplish the task successfully and without interference. In contrast, the symbolic cueing task is much harder as it involves additional cognitive load and thus requires more effortful processing. Consequently, interference effects were boosted in participants whose resources are limited and therefore depleted sooner by the more challenging task (see Wishhart et al., 2000 for similar argument). This argument is also in line with the observation that deficits in interlimb coordination for older adults can only reliably observed in conditions which are more difficult and require intense attentional and executive control (Bangert, Reuter-Lorenz, Walsh, Schachter, & Seidler, 2010). Interestingly, Bangert et al. (2010) also found that the deficits in the more difficult coordination task correlated with the working memory performance of their older participants suggesting that, more generally, a decline in cognitive measures may relate to deficits in sensorimotor control as task demands increase (see also Baltes & Lindenberger, 1997).

It is important to point out that the observed difference in the size of the interference effect could not be explained by either a simple speed–accuracy trade-off (i.e., reduced interference effects due to reduced accuracy in movement planning and control) as movements were similarly accurate for all our participant groups. The only effect we observed with regard to accuracy was the finding that movements were slightly less accurate for asymmetric as compared to symmetric movements which is congruent with the bimanual coordination literature (e.g., Wenderoth, Debaere, Sunaert, & Swinnen, 2005). Similarly, there was no indication that smaller interference effects during movement initiation were associated with longer movement times or the capacity to decouple both hands. It has been argued previously that whether or not a movement interference effect can be observed during movement planning may depend on the overall duration of the movements as longer MTs may increase the likelihood of movement amplitudes being fully specified during movement execution rather than during movement initiation (Heuer & Klein, 2006). In our experiment, we observed not only longer MTs for symbolic cueing conditions in which the movement interference effect was actually much more prominent but also, as discussed above, a positive correlation between MTs and interference effects in the direct cueing condition. This latter finding can be accounted for by the fact that interference effects during direct cueing only ever really occurred in the older adults who were also found to move more slowly. In other words, the differences in the size of the movement interference effect between our participant groups cannot be attributed to their differences in MTs as those individuals who executed their movements more quickly (i.e., musicians) showed overall smaller interference effects than individuals that moved more slowly (i.e., older adults). Finally, Stelmach et al. (1988) found that during direct cueing, older adults tended to show a more pronounced increase in MTs for asymmetric movements than for younger adults. In our study, this seemed to be the case only for the symbolic cueing condition, but was not observed during direct cueing (see Fig. 5a).

As highlighted above, the prevailing view is that interference arises at both the motor and cognitive levels. Since our participants’ groups are likely to have varied in both their cognitive in motor resources, our findings do not provide new insights into the relative contribution of those factors. However, we would like to argue that the distinction between motor and cognitive resources and their relative contributions to bimanual interference might actually be of little use to understand the complexity of motor coordination tasks—as they are essentially inseparable. For example, we observed that there were overall differences in the RTs of our participant groups with musicians initiating their movements the fastest and older adults being the slowest. At first sight, this finding could be (and has often been) interpreted as indicating a decline in the motor processes with aging (e.g., Stelmach et al., 1988) and increase in motor expertise with instrumental practice (e.g., Hughes & Franz, 2007). However, one might just as well argue that movement initiation times depend on the general information processing speed of the system which represents a core cognitive capacity. It is well known from studies investigating hand movements under dual-tasking conditions (i.e., requiring the execution of a simultaneous cognitive task) that RTs tend to increase as additional task demands are added (e.g., Hesse, Schenk, & Deubel, 2012; Liu, Chua, & Enns, 2008). In fact, the observation that RTs of older adults increased proportionally more under the dual-task requirements of the symbolic cueing condition provides a clear demonstration for the fact that RTs cannot be regarded a mere measure of the capacity of the motor system but are strongly affected by the cognitive demands of the task. Consequently, RTs will vary with the available cognitive processing resources of an individual. Based on this reasoning, we suggest that the distinction between cognitive and motor interference is quite a theoretical one and that, to understand bimanual coordination constraints, it is more relevant to look at the specific task demands and the resources required and available to an individual to deal with them. This view is clearly supported by our regression results and is also well in line with the current understanding that cognition penetrates action and that motor control is very closely linked to perceptual and cognitive processes (e.g., Oliveira & Ivry, 2008; Swinnen & Wenderoth, 2004).

In conclusion, our study provides first evidence that the interference effect during bimanual movement preparation is substantially modulated not only by task demands, but also by the processing resources available to an individual. In particular, we showed that both the general processing speed well as the available processing capacity are reliable predictors for the size of the bimanual interference effect during symbolic cueing. Our findings suggest that in order to understand the occurrence of the bimanual interference effect, it is not sufficient to focus merely on the differences between cueing conditions but also necessary to consider task demands in relation to participants’ available processing resources. The differential contributions of both factors and their precise interaction remain to be clarified.
